# Draft Genome Sequence of “*Candidatus* Phytoplasma oryzae” Strain Mbita1, the Causative Agent of Napier Grass Stunt Disease in Kenya

**DOI:** 10.1128/genomeA.00297-16

**Published:** 2016-04-21

**Authors:** Anne Fischer, Ivette Santana-Cruz, Lillian Wambua, Cassandra Olds, Charles Midega, Matthew Dickinson, Praphat Kawicha, Zeyaur Khan, Daniel Masiga, Joerg Jores, Bernd Schneider

**Affiliations:** a International Centre of Insect Physiology and Ecology, Nairobi, Kenya; b International Livestock Research Institute, Nairobi, Kenya; c Institute for Genome Sciences, Baltimore, Maryland, USA; d School of Biological Sciences, University of Nairobi, Nairobi, Kenya; e Washington State University, Pullman, Washington, USA; f Faculty of Science, The University of Notthingham, Sutton Bonington Campus, Sutton Bonington, Leicestershire, United Kingdom; g Faculty of Natural Resources and Agro-industry, Chalermphrakiat Sakon Nakhon Province Campus, Kasetsart University, Sakon Nakhon, Thailand; h Institute of Veterinary Bacteriology, University of Bern, Bern, Switzerland; i Julius Kühn-Institut, Institut für Pflanzenschutz in Obst- und Weinbau, Dossenheim, Germany

## Abstract

Phytoplasmas are bacterial plant pathogens with devastating impact on agricultural production worldwide. In eastern Africa, Napier grass stunt disease causes serious economic losses in the smallholder dairy industry. This draft genome sequence of “*Candidatus* Phytoplasma oryzae” strain Mbita1 provides insight into its genomic organization and the molecular basis of pathogenicity.

## GENOME ANNOUNCEMENT

Phytoplasmas are bacteria that parasitize the plant phloem tissue causing a wide range of symptoms, like yellowing, stunting, little leaves, witches’ brooms, virescence, and phyllody (1). They are transmitted by insect vectors and more than 1,000 plant species have been described as host plants, many of agricultural importance. Current phytoplasma classification is based on restriction fragment length polymorphism (RFLP) patterns of their 16SrDNA sequence (2). Because phytoplasmas are unculturable in axenic media, their molecular characterization has been hampered and only five phytoplasma genomes have been deposited in public databases to date (3–7). Napier grass, or elephant grass (*Pennisetum purpureum*), is the major fodder grass for the dairy industry in eastern Africa. Napier grass stunt disease (NSD) severely impairs the growth of Napier grass, with losses of up to 70% biomass in infected plants. In Kenya and Uganda, NSD is caused by a phytoplasma belonging to group 16SrXI (“*Candidatus* Phytoplasma oryzae” or rice yellow dwarf) (8, 9). We report here the first draft genome of Napier grass phytoplasma strain Mbita1.

Infected Napier grass plants were collected at the International Centre of Insect Physiology and Ecology (ICIPE) Mbita Research Station, western Kenya. A pulse-field-electrophoresis gel was done with a pilot DNA extract, which allowed the genome size of the Napier grass phytoplasma to be estimated to approximately 550 kbp. Afterward, bacterial DNA was further extracted from plant leaves using a Cs-Cl gradient, purified, and the genome was sequenced using the Illumina Hiseq sequencing platform. Illumina raw reads were split into 31 data sets. Each set was assembled independently using the Celera Assembler v8.2 at 150× coverage. Resulting contigs (approximately 30 contigs per assembly) were overlapped using Minimus (10) to find potential gaps in coverage from the independent assemblies. The final assembly resulted in 28 contigs and a genome size of 533,195 total bp (16 contigs > 10 kbp adding up to 455,349 bp). The 16S rRNA operon was absent from the assembly, due to low coverage in the region. We therefore amplified the 16S rRNA operon using PCR. Sanger sequencing of the PCR products confirmed that Napier grass phytoplasma belonged to the group 16SrXI. The average G+C content of the reported genome was 19.3%, which is the lowest value reported for a phytoplasma species to date, as the G+C content for other species ranges between 21.4% and 27.3%. Open reading frames (ORFs) were predicted using Prodigal 2.50. A total of 465 ORFs and 10 tRNAs were obtained. Functional annotation was produced by the Institute for Genome Sciences Annotation Engine (11), http://ae.igs.umaryland.edu/cgi/index.cgi. The average gene length was 888 bp. Among the ORFs, we identified the immunodominant encoding genes *imp*, *secA*, and *secY*, which are essential to the Sec protein translocation system. We also found one hflB gene and three of its truncated copies.

This annotated genome sequence is the first from the “*Candidatus* Phytoplasma oryzae” species. It will give insight into the genetic diversity of phytoplasmas and further our understanding of molecular pathogenicity mechanisms.

### Nucleotide sequence accession numbers.

This whole-genome shotgun project has been deposited at DDBJ/ENA/GenBank under the accession number LTBM00000000. The version described in this paper is version LTBM01000000. The raw reads are available under accession number SRP069757.
